# Opposite Roles of δ- and μ-Opioid Receptors in BACE1 Regulation and Alzheimer’s Injury

**DOI:** 10.3389/fncel.2020.00088

**Published:** 2020-04-30

**Authors:** Yuan Xu, Feng Zhi, Gianfranco Balboni, Yilin Yang, Ying Xia

**Affiliations:** ^1^Shanghai Key Laboratory of Acupuncture Mechanism and Acupoint Function, Department of Aeronautics and Astronautics, Fudan University, Shanghai, China; ^2^Modern Medical Research Center, The Third Affiliated Hospital of Soochow University, Changzhou, China; ^3^Department of Neurosurgery, The First People’s Hospital of Changzhou, Changzhou, China; ^4^Department of Life and Environment Sciences, University of Cagliari, Cagliari, Italy

**Keywords:** Alzheimer’s disease, BACE1, Aβ peptides, δ-opioid receptor, μ-opioid receptor

## Abstract

Alzheimer’s disease (AD) is characterized by amyloid plaques and neurofibrillary tangles. Substantial evidence for AD pathogenesis suggests that β-site APP cleaving enzyme 1 (BACE1) and γ-secretase enzyme initiate the amyloidogenic pathway and produces toxic Aβ peptides that prone to aggregate in the brain. Therefore, the inhibition of BACE1 expression and function is an attractive strategy for AD therapy. In the present work, we made the first finding that activating δ-opioid receptors (DOR) with a specific DOR agonist significantly attenuated BACE1 expression and activity in the highly differentiated PC12 cells with mimicked AD injury, while the application of DOR inhibitor naltrindole reversed the UFP-512 effects, and even caused a major increase in BACE1 expression and activity as well as Aβ42 production in physiological conditions. Knocking-down DOR also enhanced BACE1 protein expression and its activity for APP processing, associating with a significant increase in Aβ42 production. In sharp contrast, activation of μ-opioid receptor (MOR) with DAMGO greatly promoted BACE1 expression and activity with an acceleration of APP cleavage, thus contributing to increased Aβ42 production. DADLE, a less selective DOR agonist that may bind to MOR, had no stable inhibitory effect on BACE1. Similar results were also found in APP mutant (APPswe) SH-SY5Y cell line, providing further validation of the DOR action on BACE1 regulation. Our novel data demonstrated entirely different roles of DOR and MOR in the regulation of BACE1 expression and activity with DOR being neuroprotective against AD injury. These findings provided a novel clue for new strategies of AD therapy via targeting endogenous opioid receptors.

## Introduction

Alzheimer’s disease (AD) is characterized by a progressive loss of memory and cognition, which is the main cause of dementia and one of the great health problems of the 21st century. Indeed, dementia seriously affects almost 50 million people worldwide, and AD is the most well-known form of dementia, accounting for 50–75% of all cases (Cheignon et al., 2018). Two main pathological hallmarks of AD are amyloid plaques and neurofibrillary tangles. It has been widely accepted, though partially challenged now, that the deposition of Amyloid-β triggers neuronal dysfunction and death in the brain, i.e., so-called the amyloid cascade hypothesis (Ballard et al., 2011). Amyloid-β peptides result from the sequential cleavage of APP, a type I integral membrane glycoprotein. There are two pathways lead to either non-amyloidogenic or amyloidogenic processing of this protein. In the non-amyloidogenic pathway, APP is cleaved by α-secretase and γ-secretase in its amyloid-β domain, producing soluble p3 peptides and the APP intracellular domain (AICD). The replacement of α-secretase with β-secretase (BACE1) changes the pathway to the amyloidogenic pathway, which releases amyloid-β peptides as well as the AICD fragment. The amyloid-β peptides with longer lengths such as Aβ42 are more prone to aggregation and more toxic (Canter et al., 2016).

Current medical management has a limited therapeutic effect on the quality of life for individuals with AD. Therefore, new treatments to prevent, delay, and cure AD are urgently needed. Several new approaches to treat AD have been proposed, most of them target Aβ. Among these, the development of specific inhibitors of BACE1 is an attractive prospect by fundamentally attenuating the production of Aβ in the first place (Scheltens et al., 2016).

Opioid receptors are widely distributed throughout the central nervous system, including the cortex and hippocampus, the major brain regions essential to cognition, learning and memory (Xia and Haddad, 1991, 2001; Erbs et al., 2015; Xia, 2015; Pellissier et al., 2018). Substantial data from our laboratory and other independent groups have demonstrated that δ-opioid receptor (DOR), rather than μ-opioid receptor (MOR), is an important neuroprotector in various injuries (He et al., 2013; Xia, 2015). DOR activation can stabilize brain ionic homeostasis (Chao et al., 2012; He et al., 2013; Lee et al., 2018) and inhibit hypoxic/ischemic neuroinflammation by regulating a PKC-dependent and a PKA-independent signaling pathway, increasing Nrf2 translocation and regulating PINK1-mediated mitochondrial homeostasis (Yang et al., 2009; He et al., 2013; Wang et al., 2014; Cao et al., 2015; Qiu et al., 2019; Xu et al., 2019). Since emerging evidence suggests that the pathogenesis of AD involves mitochondrial dysfunction, oxidative stress damage, calcium homeostasis and inflammation (Scheltens et al., 2016; Cheignon et al., 2018; Lee et al., 2018), we have proposed that opioid receptors may play a role in the pathology of AD, including regulations of the BACE1-mediated Aβ production with δ-opioid receptor (DOR) being neuroprotective.

However, a few studies seem to suggest that DOR contributes to the pathogenies of AD through promoting the APP processing. They argued that DOR activation enhanced β- and γ-secretase activities to mediate a specific processing of APP to Aβ, while DOR antagonism ameliorated Aβ pathology and Aβ-dependent behavioral deficits (Ni et al., 2006; Teng et al., 2010). In their work, a relatively non-specific opioid ligand, DADLE, was used as a DOR agonist in non-neuronal cell line (HEK293T cells). Since DADLE may bind to MOR and MOR may have a different role in certain neural functions (Lutz and Kieffer, 2013; Xia, 2015; Shrivastava et al., 2017; Wang et al., 2018) and since non-neuronal cells may react to AD stress in a different way, it is important to adopt more specific methodologies in neuron-like cells to yield more reliable conclusions in terms of the role of DOR in the pathology of AD.

In this study, we used a specific and potent DOR agonist and compared its effect with DADLE on the BACE1-mediated Aβ production and cell injury in highly differentiated PC12 cells. Moreover, we investigated the role of MOR activation in the BACE1-mediated Aβ production and cell injury in parallel. Our outcome data form the first evidence that DOR and MOR have entirely different roles in the BACE1 regulation and Aβ1–42 oligomer induced injury with DOR being neuroprotective.

## Materials and Methods

### Chemicals and Reagents

Amyloid β1–42 peptide were purchased from Sigma-Aldrich (Cat: SCP0038, respectively, Bay St. Louis, MS, United States). UFP-512, a highly selective DOR agonist, was synthesized by our research partner (Aguila et al., 2007). Naltrindole hydrochloride, a DOR antagonist; DADLE, a DOR unspecific agonist; and DAMGO, a MOR agonist were all purchased from Tocris Bioscience (Cat: 0740, 3790, 1171, Bristol, United Kingdom). Cell Counting Kit-8 was from Beyotime, Co. (Cat: C0039, Shanghai, China). BACE1 Rabbit polyclonal antibody were purchased from Proteintech (Cat:12807-1-AP, Rosemont, IL, United States). Anti-β-actin antibody and APP rabbit antibody were purchased from Cell Signaling Technology (Cat: 4272, 76600S, Danvers, CO, United States). BACE1 Activity Kit were purchased from BioVision (Cat: K388-100, Milpitas, CA, United States). Human/Rat β Amyloid(42) ELISA Kit were obtained from Wako (Cat: 292-64501, Osaka, Japan).

### Cell Cultures and Cell Treatments

An original highly differentiated rat PC-12 cell line was obtained from the Type Culture Collection of the Chinese Academy of Sciences, Shanghai, China. APP mutant (APPswe) SH-SY5Y cell were built by Fubio, Suzhou, China. The cells in the control group were incubated at 37°C in a humidified incubator with 5% CO_2_. To mimic AD cell injury, PC-12 cells were exposed to 20 μM of Aβ1–42 oligomer for 48 h after cell attachment. UFP-512 (5 μM), a specific and potent DOR agonist, and naltrindole (1 μM), a DOR antagonist (Lutz and Kieffer, 2013; Scheltens et al., 2016; Xu et al., 2019), were applied to the culture medium in the subsequent experiments based on the preliminary results. Two concentrations of DADLE (1, 5 μM), a relatively non-specific DOR agonist and DAMGO (1 and 5 μM), a μ-opioid receptor (MOR) agonist (Ni et al., 2006; Lutz and Kieffer, 2013; Chen et al., 2014, 2019), were applied to the culture cells in the same way.

### Cell Viability Assay

Cell viability was measured by CCK8 kit. Exponentially growing cells were plated at 5000 cells/well in a 96-well plate. A blank control was set with the well no cells but containing culture medium. After appropriate duration of drug treatments, the original medium was removed and 100 μl of fresh culture medium was added. 10 ul of CCK8 reagent per well was applied and the cells were incubated another 2 h for sufficient reaction. The absorbance was measured at the wavelength of 450 nm using a microplate reader.

### Fluorometric Secretase Activity Assay

BACE1 secretase assay kit was used to measure BACE1 activity according to the manufacturer’s manual. In brief, the cell lysate fractions were extracted by BACE1 extraction buffer, and the samples were incubated with the secretase-specific substrate conjugated to a fluorescent reporter. An EDANS standard curve was set to evaluate the relative BACE1 activity. The wavelength at Ex/Em = 345/500 nm was detected in a kinetic mode for 10–60 min at 37°C on a multi-well spectrophotometer. The sample BACE1 activity was calculated as follows:

$$S\,a\,m\,p\,l\,e\,\;\,B\,A\,C\,E\,1\,\;\,A\,c\,t\,i\,v\,t\,y=\frac{B}{\Delta \,t\times V}\times D=m\,U/m\,l$$

Where: B is the EDNAS amount from Standard Curve(pmol)

Δt is the reaction time(min)

V is the sample volume added into the reaction well(μl)

D is the dilution factor.

### Aβ42 Measurements

The Aβ42 levels were determined using a Human/Rat β Amyloid (42) ELISA kit. Following the manufacturer’s instruction, the medium sheds and the cell lysates were collected and quantified. The samples and standards incubated in the wells for overnight at 4°C. Then the HRP conjugated antibody was added and TMB solution was used to start the HRP reaction at RT in the dark. After the reaction was terminated by stop solution, the absorbance was read at 450 nm with a multi-well microplate reader. Aβ42 concentrations were calculated based on the standard curve.

### Western Blotting

Cells were lysed using the lysis buffer containing 0.5% 100 mM PMSF, 0.1% protease inhibitor, and 1% phosphatase inhibitor (KeyGEN Biotec, Cat: KGP2100, Nanjing, China). The protein concentration was determined by the BCA protein assay kit according to the manufacturer’s protocols. Equal amounts of protein samples were diluted in a 5x loading buffer and run in 10–12.5% SDS-PAGE electrophoresis. Then proteins were transferred to hydrophobic polyvinylidenedifluorid (PVDF) membranes, and the membranes were blocked and incubated with certain mAbs. HRP conjugated secondary antibodies were used to detect the specific protein expression and it was visualized by chemiluminescence exposure using Western Lightening^®^ Chemiluminescence Reagent Plus (Perkin-Elmer, Boston, MA, United States). Quantitation was performed by densitometry using the NIH Image program (Image J).

### BACE1 mRNA Quantification

To measure the changes in the BACE1 mRNA content, total RNA from the cells was isolated using trizol reagent according to the manufacturer’s instruction. In brief, the extracted RNA (1 μg) was used for the template in reversed transcription. RT-PCR was performed with the SYBR Select Master Mix (Applied Biosystems, Foster City, CA, United States). The primers designed are listed as follows:

BACE1 mRNA primers:

Sense 5′ AGACGCTCAACATCCTGGTG 3′

Anti-Sense 5′ CCTGGGTGTAGGGCACATAC 3′

Ribosomal mRNA primers:

Sense 5′ CAGAAGGACGTGAAGGATGG 3′

Anti-Sense 5′ CAGTGGTCTTGGTGTGCTGA 3′

The target mRNA levels were calculated depending on the Ct value of the target cDNA and the reference cDNA. The relative quantification of target mRNA were adjusted to the control.

### siRNA Transfection

The siRNA oligonucleotides were synthesized by Genepharma, Co. (Shanghai, China). The sequences were designed as follows:

Negative control siRNA:

5′-UUC UCC GAA CGU GUC ACG UTT-3′

DOR siRNA (rat):

5′-GGC UGU GCU CUC CAU UGACUU-3′

DOR siRNA (human):

5′-GCCAAGCUGAUCAACAUCUTT-3′

PC12 cells and APPswe SH-SY5Y cells were transfected with DOR small interfering RNA (rat) constructs or DOR siRNA (human) respectively. The negative control were established following the manufacturer’s instructions (Genepharma, Co., Shanghai, China). The siRNA compounds were diluted in Opti-MEM and then mixed at a 1:1 ratio with diluted Lipofectamine in Opti-MEM. The mixture was added directly to the culture medium for 20-min stabilization. After 6-h incubation, the medium containing siRNA was removed and the fresh medium was added. Successfully transfected PC12 cells were used for the follow-up experiments.

### Statistical Analysis

All data are presented as means ± SEM and the independent experiments number performed for each measurement is at least three. Statistical analysis was processed with one-way ANOVA followed by Bonferroni’s multiple comparison tests (Prism 5, GraphPad Software, La Jolla, CA, United States).

## Results

### DOR Inhibition and MOR Activation Aggravated Aβ1–42 Oligomer Induced Cell Injury

**FIGURE 1:**
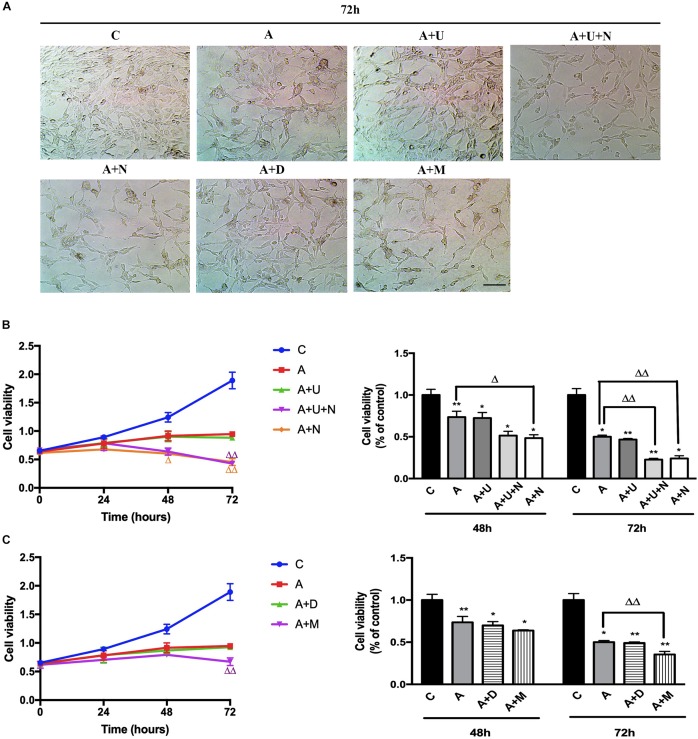
DOR inhibition or MOR activation aggravated Aβ1–42 oligomer induced cell injury. The experiments were conducted on highly differentiated PC12 cells (*N* = 3 in each group). The cell viability were measured every 24 h using CCK8 kit. C: normal control. A: Aβ1–42 oligomer (20 μM) to mimic AD cell injury (Aβ1–42 injury or AD injury). A + U: DOR activation with UFP-512 in AD injury. A + U + N: A + U plus DOR inhibition with naltrindole. A + N: DOR inhibition with naltrindole in AD injury. A + D: DADLE (1 μM) treatment in AD injury. A + M: MOR activation with 1 μM DAMGO in AD injury. **(A)** DOR/MOR induced morphologic changes in PC12 cells. Note that Aβ1–42 oligomer induced cell injury and the administration of DOR antagonist naltrindole or MOR agonist DAMGO aggravated such injury. Morphologically, it was characterized by a reduction in cell density, decrease of synapses. Scale bar = 100 μm in panel **(A)** for all groups. **(B)** Cell viability and DOR-mediated alternations in cell proliferation under AD injury. *p < 0.05, **p<0.01 vs. **(C)**; ^Δ^ *p*<0.05, ^Δ^ ^Δ^ *p* < 0.01 vs. **(A)**. Note that Aβ1–42 oligomer administration largely decreased the cell viability, and DOR inhibition further aggravated the cell injury. **(C)** Effects of DADLE and DAMGO on cell viability in Aβ1–42 injury. *p < 0.05, **p<0.01 vs. **(C)**; ^Δ^ ^Δ^ *p* < 0.01 vs. **(A)**. Note that MOR activation greatly aggravated the cell viability at 72 h after Aβ1–42 oligomer administration.

Aβ oligomers have been well-linked to AD pathogenesis in animal models and human beings (Selkoe and Hardy, 2016; Jeong, 2017) and Aβ oligomer injury is now widely used to mimic AD injury in cell models (Geng et al., 2013; Balducci and Forloni, 2014; Morroni et al., 2016). In this work, we induced the AD injury by treating the highly differentiated PC12 cells with 20 μM Aβ1–42 oligomer (Aβ1–42 injury or AD injury). After Aβ1–42 oligomer exposure for 48 h, the cell viability showed a significant reduction by 26.4% vs. the control group (*p* < 0.01, Figure 1). The cell injury was worsen with the increase in exposure time. At 72 h, the cell viability was reduced by 50% (vs. 100% of the control group, *p* < 0.05, Figure 1). Morphologically, the cell density was significantly reduced and the synapses of the PC12 cells was decreased to some extent.

Then, we applied DOR specific agonist UFP-512 (5 μM) and DOR inhibitor naltrindole (1 μM) to the PC12 cells to examine the effects of DOR on AD injury. The application of UFP-512 did not cause any significant change in the cell viability (Figure 1B) as well as the cell density (Figure 1A). However, the addition of DOR antagonist naltrindole greatly aggravated Aβ1–42 injury. The measurements at 48 h after the treatments showed a 25.1% decrease in the cell viability (from 73.6% in the group of Aβ1–42 oligomer alone to 48.5% in the group with Aβ1–42 oligomer plus naltrindole, *p* < 0.05, Figure 1A-right panel). After prolonging the exposure time to 72 h, the inhibition of DOR induced more severe cell damage. Under the condition of Aβ1–42 injury, either UFP-512 plus naltrindole or naltrindole alone caused much more cell injury compared to that of Aβ1–42 oligomer alone (*p* < 0.01, Figure 1A-right panel).

Furthermore, we examined the effects of DADLE, a much less selective DOR agonist than UFP-512, and MOR agonist DAMGO on cell viability. As Figures 1A,C depicted, DADLE did not significantly alter the cell injury induced by Aβ1–42 oligomer, similar as UFP-512. In contrast, the cell viability and cell density were progressively reduced with the duration of DAMGO treatments, with a decrease by 13.4% after 48 h and a decrease by 29.1% after 72 h vs. Aβ1–42 injury group (*p* < 0.01, Figure 1B).

The above results suggest that DOR and MOR have differential effects on AD injury in PC12 cells.

### DOR Activation and MOR Activation Differentially Altered BACE1 Activity

**FIGURE 2:**
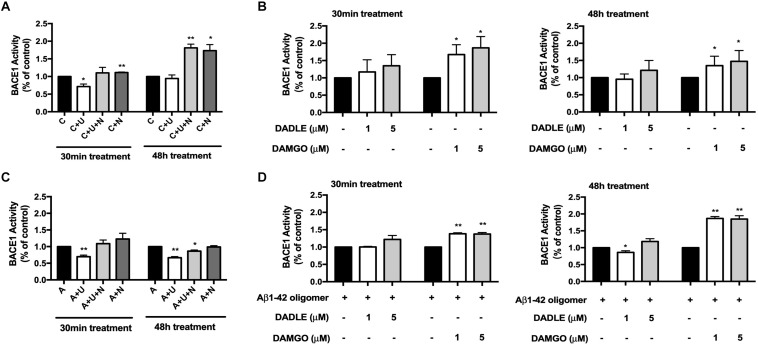
DOR inhibition or MOR activation enhanced BACE1 activities in normal conditions, while DOR activation attenuated BACE1-mediated cleavage activities in Aβ1–42 injury. BACE1 activities were examined at 30 min and 48 h after the drug treatment (*N* = 3 in each group). C: control. C + U: DOR was activated by UFP-512. C + U + N: PC12 cells were treated with UFP-512 plus naltrindole at the same time under normal conditions. C + N: DOR was inhibited by naltrindole. A: PC12 cells were treated with 20 μM Aβ1–42 oligomer to mimic the AD injury. A + U: DOR was activated by UFP-512 under AD injury. A + U + N: PC12 cells were treated with UFP-512 plus naltrindole together under AD injury. A + N: DOR was inhibited by naltrindole under AD injury. **(A)** DOR-mediated regulation of BACE1 activities in the physiological condition. *p < 0.05, **p<0.01 vs. **(C)**. Note that 30-min treatment with DOR agonist UFP-512 significantly decreased BACE1 activities, whereas the application of DOR antagonist naltrindole enhanced BACE1 activities at both 30 min and 48 h after the treatment. **(B)** Effects of DADLE and DAMGO on BACE1 activity in normal conditions. *p < 0.05 vs. **(C)**. Note that DADLE did not induce any significant change in BACE1 activity, while MOR agonist DAMGO significantly upregulated BACE1 activities. **(C)** Effects of DOR agonist UFP-512 and antagonist naltrindole on BACE1 activity under AD injury. The PC12 cells were treated with 20 μM Aβ1–42 oligomer for 48 h to mimic AD injury. UFP-512 and naltrindole were simultaneously or separately applied to these cells for 48 h or at the last 30 min of Aβ1–42 oligomer exposure. *p < 0.05, **p < 0.01 vs. **(A)**. Note that DOR activation by UFP-512 significantly reduced BACE1 activities, whereas its inhibition with naltrindole reversed such effect. **(D)** Effects of MOR activation on BACE1 activity under AD injury. The cells with AD injury were exposed to DADLE or DAMGO at 1–5 μM. *p < 0.05, **p < 0.01 vs. **(A)**. Note that the BACE1 cleavage activity was significantly reduced by DADLE at 1 μM after 48-h treatment, but largely enhanced by the application of DAMGO at 1 or 5 μM.

The accumulation of Aβ triggers neuronal dysfunction and death in the brain (Scheltens et al., 2016), while the generation of Aβ involves APP cleavage by β secretase (BACE1). We therefore investigated if DOR inhibition or MOR activation caused any changes in BACE1 activity. As shown in Figure 2A, 30-min treatment of DOR agonist UFP-512 remarkably decreased BACE1 activity by 28.4% (*p* < 0.05, Figure 2A). At 48-h of UFP-512 treatment, however, there was no appreciable change in BACE1 activity as compared to the control group. Interestingly, DOR antagonist naltrindole, significantly enhanced BACE1 activity at both 30 min and 48 h of naltrindole exposure (111.3 vs. 100% of the control group at 30 min, *p* < 0.01, and 173.4 vs. 100% of the control group at 48 h, *p* < 0.05, Figure 2A). Even in the existence of UFP-512, naltrindole still increased BACE1 activity by 10.3% at 30 min and 81.1% at 48 h as compared to the control group (*p* < 0.01, Figure 2A).

Similarly, DADLE- and DAMGO-mediated alternations in BACE1 activity were evaluated using the BACE1 activity kit. We applied DADLE and DAMGO at 1 and 5 μM to the cells and lasted for 30 min or 48 h. The results showed that 1 μM DADLE did not cause a major impact on BACE1 activities, but 5 μM DADLE slightly enhanced BACE1 activity at both 30 min and 48 h after DADLE treatment (Figure 2B). In sharp contrast to DADLE, DAMGO significantly increased BACE1 activities in the concentrations of either 1 or 5 μM and at both 30 min and 48 h after DAMGO treatment (*p* < 0.05 vs. the control, Figure 2B).

Furthermore, we compared BACE1 activities after DOR vs. MOR activation in the AD cell model with Aβ1–42 oligomer treatment. As shown in Figure 2C, DOR activation with UFP-512 greatly reduced BACE1 activity by 30.2% after 30-min treatment (*p* < 0.01 vs. the control, “A” group) and 33.7% vs. after 48-h treatment (*p* < 0.01 vs. the control, “A” group) (Figure 2C), whereas the addition of DOR antagonist naltrindole reversed the changes. In the same condition, DADLE had no appreciable effect on the activity of BACE1 except for a slight decrease in BACE1 activity after 48-h treatment of 1 μM DADLE (+13.9%, *p* < 0.05, Figure 2D). In sharp contrast to the DOR agonists, MOR agonist DAMGO significantly increased the activity of BACE1 under AD stress (+30%, *p* < 0.01, Figure 2D) in the exactly same condition.

These data suggest that DOR and MOR regulate BACE1 activities in an opposite direction under both normal condition and AD injury.

### DOR Inhibition and MOR Activation Induced BACE1 Upregulation Were Associated With an Increase in Aβ42 Production and Release

**FIGURE 3:**
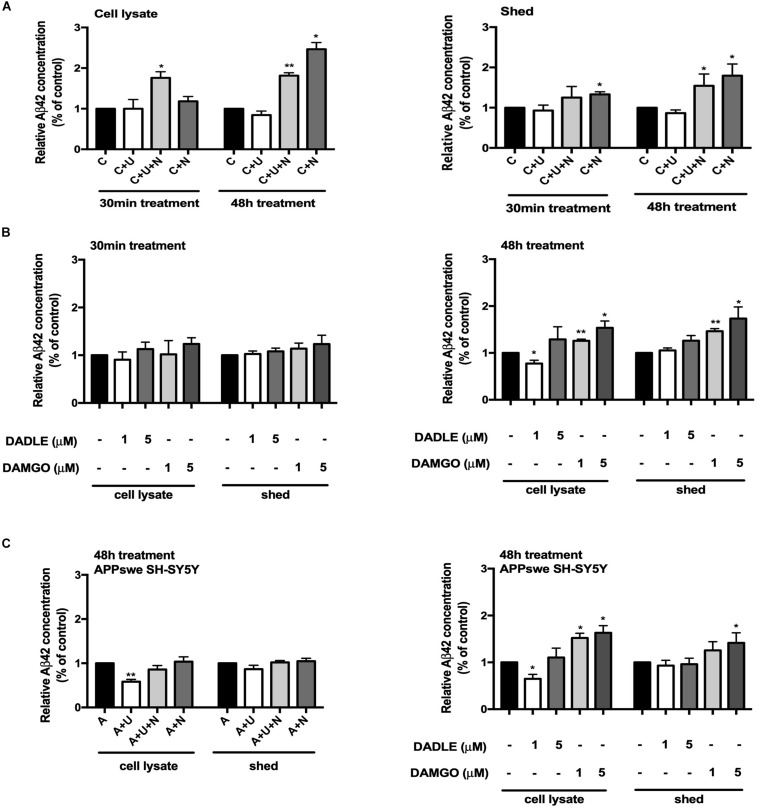
DOR and MOR differently regulated Aβ42 production and release. Aβ42 concentrations were measured in the cell lysate and medium shed (*N* = 3 in each group). **(A)** Effects of DOR activation and inhibition on Aβ42 production and release in PC12 cell line. *p < 0.05, **p<0.01 vs. **(C)**. Note that DOR antagonist naltrindole significantly increased the Aβ42 levels in both cell lysate and medium shed though UFP-512 did not induce any significant change. **(B)** DADLE- and DAMGO-induced changes in Aβ42 production and release in PC12 cell line. The PC12 cells were treated with DADLE or DAMGO at 1 and 5 μM. *p < 0.05, **p < 0.01 vs. **(C)**. Note that 30-min cell exposure to DADLE or DAMGO did not cause a significant change in the level of Aβ42 as well as its release. After prolonging the exposure time to 48 h, the Aβ42 production was slightly reduced by 1 μM DADLE treatment in the cell lysate portion, whereas the application of DAMGO remarkably increased the levels of Aβ42 in both cell lysate and medium shed. **(C)** The activation of DOR and MOR adversely regulated Aβ42 production in APPswe SH-SY5Y cell model. *p < 0.05, **p < 0.01 vs. **(A)**. Note that the 48-h exposure of UFP-512 to APPswe SH-SY5Y cells largely decreased Aβ42 production in cell lysate portion, but not in medium shed, while the addition of DOR antagonist naltrindole reversed such changes. Low concentration of DADLE also significantly attenuated Aβ42 production in APPswe SH-SY5Y cell model. In the contrast, DAMGO remarkably enhanced Aβ42 production and release with the concentration increased.

Since Aβ42 is more prone to aggregate in the brain, the initial deposition always begins from Aβ42, but not other Aβ product (Ni et al., 2006; Ballard et al., 2011). We therefore investigated whether the alterations in BACE1 activity after opioid receptor activation/inhibition lead to any change in the production of Aβ. Using a high sensitive ELISA kit to detect the C-terminal portion of Aβ42, we found that DOR activation with UFP-512 did not significantly change the Aβ42 production, but adding its antagonist naltrindole or applying naltrindole alone to the cell medium greatly increased the level of Aβ42, especially after 48-h treatment (from 100% of the control group to 181.4% of the “C + U + N” group in the cell lysate portion, *p* < 0.01, from 100% of the control group to 246.3% of the “C + N” group in the cell lysate portion, *p* < 0.05, from 100% of the control group to 154.4% of the “C + U + N” group in the medium shed portion, *p* < 0.05, from 100% of the control group to 179.6% of the “C + N” group in the medium shed portion, *p* < 0.05, Figure 3A).

With DADLE or DAMGO treatment, there was no appreciable change in Aβ42 production after 30-min treatments. The treatment with DADLE at 1 μM for 48 h led to a significant decrease in Aβ42 level in cell lysate, but not in the shed portion of Aβ42. The treatment with DAMGO, especially in a higher concentration (5 μM), remarkably increased Aβ42 production in both the cell lysate and medium shed (*p* < 0.05, Figure 3B).

To further validate the effects of DOR and MOR on Aβ production and release, we used SH-SY5Y cells with an APP mutant (APPswe), which thus harbors “Swedish” mutations, a characteristic of some AD cases. These cells possess endogenous DOR, BACE1 expression and activities. As shown in Figure 3C, the stimulation of DOR with its specific agonist UFP-512 for 48 h decreased the levels of Aβ42 in the lysate portion of APPswe SH-SY5Y cells (*p* < 0.01, Figure 3C), but not significantly altered the Aβ42 concentration in the medium shed. When exposing to a low concentration of DADLE (1 μM), these cells also showed a significant reduction of Aβ42 production (*p* < 0.05, Figure 3C). In contrast, MOR activation using DAMGO induced a gradual increase in Aβ42 in both the cell lysate and medium shed (*p* < 0.05, Figure 3C).

These results suggest that the changes in DOR and MOR activities alter both BACE1 activity and Aβ42 production.

### MOR Activation Increased BACE1 mRNA and Protein in Physiologic and AD Conditions, While DOR Activation Down-Regulated BACE1 Expression in AD Injury

**FIGURE 4:**
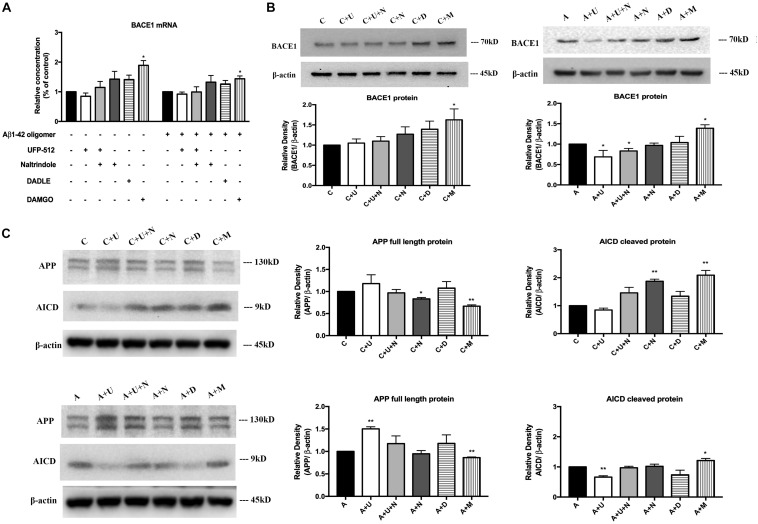
DOR activation down-regulated, but MOR activation up-regulated BACE1 expression for APP processing. BACE1 mRNA and proteins were measured in the PC12 cells in the conditions of normal control and Aβ1–42 injury (48 h). APP processing was evaluated by measuring APP full length protein and AICD. *N* = 3 in each group. *p < 0.05, **p<0.01 vs. **(C)** or **(A)**. **(A)** DAMGO-induced alternations in BACE1 mRNA in physiological condition and AD injury. **(B)** DAMGO-induced upregulation of BACE1 protein in both physiological condition and AD injury, while DOR activation specifically attenuated BACE1 expression under AD injury. **(C)** APP cleavage was associated with DOR activation and inhibition, while MOR activation significantly accelerated APP processing. Note that DOR activation had no significant effect on BACE1 mRNA expression though it tended to induce a slight reduction in both physiological and Aβ1–42 oligomer conditions. However, it significantly reduced the level of BACE1 protein in AD condition (Aβ1–42 oligomer exposure), which could be largely reversed by DOR antagonist naltrindole. In contrast, MOR activation with DAMGO significantly increased the expression of both BACE1 mRNA and protein in both physiological and Aβ1–42 oligomer conditions. Consistently, the APP cleavage was seriously interfered by DOR activation in Aβ1–42 oligomer conditions, while DAMGO accelerated APP processing in both physiological and Aβ1–42 oligomer conditions with a significant decrease in APP full length protein and a large increase in AICD, the APP cleavage product.

Next, we asked if DOR and MOR modulate BACE1 expression at mRNA and/or protein levels. The total RNA were extracted from each treated group, specific BACE1 primers were used to quantify the relative concentrations of target mRNA. Under physiologic conditions, DOR activation led to an unappreciable effect on the level of BACE1 mRNA, while the inhibition of DOR by naltrindole tended to increase BACE1 mRNA level though not significant in our sample size (Figure 4A). On the other hand, MOR activation induced a significant increase in BACE1 mRNA (from 100% in the control group to 189.2% in the group treated with 1 μM DAMGO, *p* < 0.05, Figure 4A). Similar results were observed with the treatment of DAMGO in the group of Aβ1–42 oligomer injury. DADLE also tended to slightly increase BACE1 mRNA, though the change is not significant in our sample size. We then measured the BACE1 protein expression using Western blotting. In normal conditions, the results were consistent with those of BACE1 mRNA. The application of DOR agonist UFP-512 or DOR antagonist naltrindole caused unappreciable change in BACE1 protein levels, while DADLE caused a slight increase in the level of BACE1 protein under physiological conditions. The 48-h exposure to DAMGO led to a remarkable increase in BACE1 protein in the PC12 cells (from 100% in the control group to 162.7% in the group treated with 1 μM DAMGO, *p* < 0.05, Figure 4B). When the cells were exposed to the AD insult, i.e., 48-h treatment of Aβ1–42 oligomer, UFP-512, the specific DOR agonist, significantly decreased BACE1 protein level by 31.2% (*p* < 0.05, Figure 4B) whereas the addition of naltrindole reversed the effects induced by UFP-512. The application of DAMGO largely enhanced BACE1 protein by 39.0% (*p* < 0.05, Figure 4B).

To further ascertain the effects of DOR and MOR on BACE1 regulation, we also evaluated the APP cleavage efficiency by measuring the APP full length protein and its cleavage product, AICD. We found that in physiological conditions, 48-h exposure to UFP-512 induced a slight increase in APP protein and a decrease in AICD, while the addition of DOR antagonist naltrindole reversed these change. Treating cells with naltrindole alone or treating cells with 5 μM DAMGO largely accelerated APP processing, with a significant conversion from APP full length protein to the cleavage form (*p* < 0.01, Figure 4C). Moreover, we tested the state of APP processing under AD injury and found that DOR activation with UFP-512 remarkably decreased the APP cleavage efficiency (*p* < 0.01, Figure 4C). In contrast, the DOR antagonist naltrindole abolished the effect of UFP-512 on APP processing. DAMGO kept promoting APP cleavage with a significant increase in AICD (*p* < 0.05, Figure 4C). DADLE showed an inappreciable effect on APP processing both under physiological and Aβ1–42 oligomer conditions.

Taken together, DOR activation reduced BACE1 activities and BACE1 protein especially under AD injury, while MOR activation induced an opposite effect and showed an increase in BACE1 activities as well as BACE1 protein and mRNA. The status of APP processing was differentially changed in response to DOR and MOR activation with a major alteration in BACE1 expression and activity.

### DOR Knockdown Enhanced BACE1 Expression and Activities for APP Processing and Increased Aβ 42 Production

**FIGURE 5:**
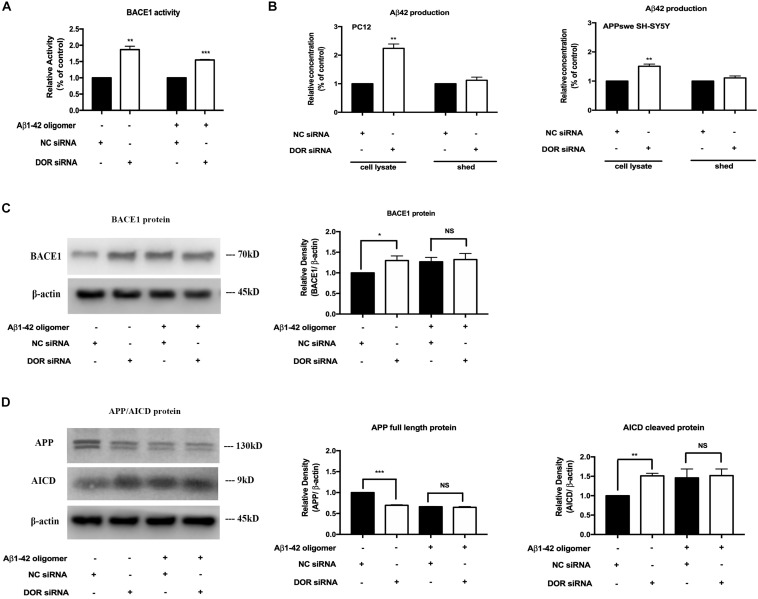
Knocking-down DOR greatly enhanced BACE1 activity and expression for abnormal APP cleavage and contributed to the Aβ42 production. The PC12 cells transfected with DOR siRNA or negative control siRNA were cultured in the conditions of normal control and Aβ1–42 oligomer for 48 h. Similarly, APPswe SH-SY5Y cells were also transfected with human DOR siRNA or negative control siRNA for Aβ42 measurement. *N* = 3 in each group. NS: not significant, *p < 0.05 vs. **(C)** or **(A)**; **p < 0.01 vs. **(C)** or **(A)**; ***p < 0.001 vs. **(C)** or **(A)**. **(A)** BACE1 activity before and after DOR knockdown. Note that DOR knockdown significantly increased BACE1 activities in both normal and AD conditions. **(B)** DOR knockdown induced effects on Aβ42 production and release in PC12 and APPswe SH-SY5Y cell models. DOR knockdown caused a sharp increase in the Aβ42 level production in the cell lysate, but not in the medium shed in PC12 cells and the same was true in APPswe SH-SY5Y cell model. **(C)** BACE1 expression was largely increased by DOR knockdown in physiological condition but not under Aβ1–42 injury. Note that DOR knockdown up-regulated BACE1 protein in physiological condition, but did not further increase BACE1 protein in the cells exposed to Aβ1–42 olimoger that already had an increased BACE1 protein. **(D)** DOR knockdown was associated with a rise in APP processing. Note that along with an increase in BACE1 expression after DOR knockdown, the transfection with DOR siRNA also significantly increased APP cleavage in the physiological condition. Aβ1–42 olimoger caused a quick cleavage of APP, which was not further enhanced by DOR knockdown.

To further ascertain the inhibitory regulation of DOR in BACE1 expression and function, we knocked down DOR expression and then examined the BACE1 expression and activities in the PC12 cells. The cells were transfected with DOR siRNA or negative control siRNA and were cultured in normal condition and that of Aβ1–42 injury. The results showed that the knockdown of DOR significantly increased BACE1 activities both under physiological and AD injury conditions (a significant increase by 86.6% vs. the control group, *p* < 0.01, a rise by 54.8% vs. “A” group after 48-h exposure, *p* < 0.001, Figure 5A). Consistently, the DOR knockdown-mediated enhancement of BACE1 activities was associated with a sharp increase in Aβ42 production by 123.9% in cell lysate (*p* < 0.01, Figure 5B), but not in medium shed. Moreover, we investigated the effects of DOR knockdown on Aβ42 production in APPswe SH-SY5Y cells. Similar results were obtained by measuring the Aβ42 level in the cell lysate and medium shed portion. DOR knockdown induced a 53.1% increase of Aβ42 production in the cell lysate portion (*p* < 0.01, Figure 5B), but did not significantly altered the concentration of Aβ42 in the medium shed.

Furthermore, we examined the BACE1 expression and APP cleavage state in the cells before and after DOR knockdown. We found that DOR knockdown remarkably up-regulated BACE1 expression and promoted APP cleavage in normoxic conditions (Figures 5C,D). In the cells with Aβ1–42 injury, there was already a large increase in BACE1 protein level and an acceleration in APP processing. In such condition, DOR knockdown could not further enhance the level of BACE1 protein and enhance APP cleavage (Figures 5C,D).

All these results strongly demonstrated that DOR antagonism or knockdown promoted the destructive APP processing by up-regulating BACE1 expression and activities, suggesting an inhibitory role of DOR in the regulation of BACE1 expression and function.

## Discussion

WE made the following major findings in this work: (1) Specifically activating DOR attenuated BACE1 expression and its cleavage activity in the highly differentiated PC12 cells with mimicked AD injury, but not in physiological condition; (2) DOR antagonism induced a significant increase in BACE1 activity for APP cleavage, and contributing to Aβ42 production; (3) Knocking-down DOR increased BACE1 expression and activity for APP processing in physiological condition with an increase in Aβ42 production in both the PC12 cells and APPswe SH-SY5Y cell models, but could not further increase BACE1 expression and APP cleavage in the AD-injured cells that already had an increase in BACE1 expression and accelerated APP processing; and (4) MOR activation aggravated the mimicked AD injury with an upregulation of BACE1 expression and activity as well as APP process. Our data strongly demonstrate that DOR plays an inhibitory role in the regulation of BACE1 expression/activity under Aβ1–42 oligomer induced injury and the opposite is true for the same opioid receptor family member MOR, suggesting DOR is neuroprotective against AD injury.

There are three major classes of opioid receptors: DOR, MOR, and KOR (kappa-opioid receptor) (Xia and Haddad, 1991, 2001; Cai and Ratka, 2012; Xia, 2015). These receptors are differentially altered in the distinct areas of AD patients’ brain in term of their expression (Mathieu-Kia et al., 2001; Xia and Haddad, 2001). Several previous reports suggest a relevance between opioid receptors and changes in AD pathology/behaviors, including alterations in cognition, hyperphosphorylated tau, Aβ production, and neuro-inflammation (Meilandt et al., 2008; Cai and Ratka, 2012). Although the specific roles of three opioid receptors in AD are not clear, a few studies (Ni et al., 2006; Teng et al., 2010) suggest that DOR is a “bad guy” favoring AD pathology. To confirm these observations for next mechanistic investigation, we conducted this work with more specific approaches on neuron-like cells.

Unexpectedly, our data draw a different conclusion, i.e., DOR is a “good guy” against AD injury. Having recognized the authenticity of the previous studies, we realized that the major difference between our results and those of others is the difference in methodologies and cell models. DADLE, a relatively non-specific DOR agonist, was used in previous work (Ni et al., 2006; Teng et al., 2010), which might activate MOR (Blurton et al., 1986; Balboni et al., 2002; Xia, 2015). In contrast to the previous work, the present study adopted a potent and specific DOR agonist UFP-512 in neuron-like cells with combinations of DOR antagonism and knockdown for the confirmation of DOR’s effect. Indeed, our parallel comparison between DADLE and UFP-512 showed that the former did not induce the same neuroprotection as UFP-512, or even exacerbated the AD injury, which was very likely due to MOR activation, one of DADLE’s dual actions. Moreover, the highly differentiated PC12 cells we used in the present study are of neuronal properties and their reactions to DOR signaling and AD injury are closer to the true actions of neurons. To further confirm our conclusions, we also used a stable APP mutant neuronal cell line, APPswe SH-SY5Y cell line that expresses endogenous DOR and BACE1. In sharp contrast, the cells used in the previous studies were HEK293T cells, non-neuronal cells.

Although numerous disrupted processes are interconnected in AD and drive the disease progresses, decades of research in genetic work on AD continue to suggest the accumulation of Aβ in the brain contributes to the loss of synapses, neurodegeneration and cell death (Canter et al., 2016). Since BACE1 is the rate-limiting enzyme in the amyloide cascade, it is of high significance to explore the linkage between opioid receptors and BACE1 regulation. Our novel data well-demonstrated that as compared to DOR, MOR has an entirely different role in the regulation of BACE1 and AD pathology. Its activation greatly enhanced BACE1 expression and activity, thus contributing to abnormal APP processing and increased Aβ42 production, aggravating AD-like injury. Meilandt et al. (2008) previously reported that the irreversible blockade of MOR with β-funaltrexamine ameliorated memory deficits induced by human amyloid precursor protein expressed in transgenic mice. Our results and their observation support each other in terms of MOR’s role in AD pathology. Since DADLE is not a specific ligand for DOR (Blurton et al., 1986; Xia, 2015), its side action on MOR is likely the reason for DADLE-induced upregulation of BACE1 expression and activity as shown in the previous reports (Ni et al., 2006; Teng et al., 2010).

BACE1 is a critical target for AD because of its irreplaceable role in the generation of Aβ. However, BACE1 also functions as a housekeeping enzyme and is involved in several processes that are necessary for proper physiology of neuronal tissue (Yan and Vassar, 2014; Barao et al., 2016; Yan, 2017). For instance, BACE1 has been shown to play an important role in synaptic plasticity and development through cleavage of its substrates (Das and Yan, 2017). Germline BACE1 knockout mice have been reported to exhibit reduced survival rate, hypomyelination, seizures, and memory deficits (Laird et al., 2005; Hitt et al., 2010; Hu et al., 2010; Barao et al., 2016). Conditional BACE1 knockout mice showed disrupted organization of axonal pathway in the hippocampus, an important region for learning and memory (Ou-Yang et al., 2018). These results indicate the importance of proper expression and function of BACE1 in the survival and function of nerve cells. It is interesting, but not surprised to us, to note that DOR activation had a marked inhibitory effect on BACE1 expression and activity in the PC12 cells with mimicked AD injury, but not in those under physiological condition. In physiological condition, the cells have sufficient DOR signals to maintain an appropriate level of BACE1 expression and activity. There is no need for additional DOR signals. The addition of limited amount of exogenous DOR agonist may not necessarily break the balance and alter BACE1 expression and function. In AD injury, however, the balance is disrupted by pathological changes with an over-production of Aβ peptides. Moreover, the redundant Aβ could be quickly degraded in the healthy cells under normal conditions, but not in AD pathogenic cells (Canter et al., 2016; Scheltens et al., 2016). The cells critically need a negative power to suppress such pathological alteration and restore normal functions. At this stage, an increase in DOR activity well meets the need of the injured cells and strengthens the inhibitory force for attenuating BACE1 expression and activity and reduces Aβ peptides. This may be the reason behind the phenomenon seen in this study.

The increased BACE1 expression and activity are one of the characteristic changes in the brain of AD patients (Scheltens et al., 2016; Koelsch, 2017). The overloading of BACE1 accelerates APP cleavage and induces an over-production of Aβ peptides. Based on our present data, there is a possibility that such pathological changes may directly or indirectly relevant to the impairment of DOR expression/function. This is because knocking down DOR alone could remarkably increase BACE1 expression and activity. Also, there is evidence showing that DOR density is largely decreased in the brain of AD patients (Mathieu-Kia et al., 2001). Therefore, we assume that restoring or strengthening DOR signaling is of great help for fighting against the pathology of AD. A strategy for DOR upregulation, either through increasing DOR expression or activity, might rebalance cellular ability to produce Aβ peptides by negatively regulating BACE1 expression and activity. Indeed, we have seen such outcomes after DOR activation in the AD-injured cells with increased BACE1 expression and activity.

The present results revealed that the concentration of Aβ42 was sharply increased by DOR knockdown in the cell lysate, but not in the shed medium of the cells. The experiments performed in APPswe SH-SY5Y showed the similar results, further validating our novel findings. It is difficult for us to explain this phenomenon at this stage. The regulatory mechanisms of Aβ42 production and transportation across the membrane are complicated and not well-understood yet. Multiple physiological and pathological factors may affect the process of Aβ42 transportation across the membrane. It is unknown if DOR knockdown, a major change in cell membrane, caused a structural change in cell membrane, dysfunction of membrane protein interaction and/or blockade of the pathway for intracellular Aβ42 movement. More studies are needed to clarify this issue.

In summary, this work made the first findings with reliable methodologies to clarify the myth about the roles of DOR vs. MOR in the pathology of AD. Specific activation of DOR by UFP-512 was neuroprotective by inhibiting BACE1 expression and activity, attenuating APP cleavage efficiency and reducing Aβ generation in AD condition, which was reversed by DOR antagonism. Interestingly, the same DOR activation did not significantly affect BACE1 expression and activity as well as Aβ production in physiological conditions. The unique role of DOR was further confirmed by the experiments with the reduction of DOR expression. DOR knockdown led to a major increase in BACE1 protein and activity, thus accelerating APP processing and increasing the cellular Aβ level in physiological condition, but did not further interfere the APP cleavage in Aβ oligomer injury. In sharp contrast, MOR activation greatly enhanced BACE1 activity/expression and contributed to AD pathology both in the physiological conditions and Aβ oligomer conditions. Our novel data suggest that DOR acts as an endogenous protector against AD injury by inhibiting BACE1 expression and activity, while the opposite is true for MOR, suggesting a possibility to develop a new strategy against AD by differentially targeting DOR and MOR.

## Data Availability Statement

The raw data supporting the conclusions of this article will be made available by the authors, without undue reservation, to any qualified researcher.

## Author Contributions

YXi initiated the project. YXu and YXi conceived and designed the experiments and wrote the manuscript. YXu performed the experiments. YXu and FZ analyzed the data. YXi, YY, and GB contributed reagents, materials, and analysis tools. YXi and YY supervised the conduct of the work. All authors read and approved the final manuscript.

## Conflict of Interest

The authors declare that the research was conducted in the absence of any commercial or financial relationships that could be construed as a potential conflict of interest. The handling Editor declared a past co-authorship with one of the authors YXi.
